# Reopenable-clip over-the-line method for closure of large perforation during esophageal endoscopic submucosal dissection

**DOI:** 10.1055/a-2176-7633

**Published:** 2023-10-06

**Authors:** Yuya Asada, Satoki Shichijo, James Weiquan Li, Noriya Uedo

**Affiliations:** 1 Department of Gastrointestinal Oncology, Osaka International Cancer Institute, Osaka, Japan; 2 Department of Gastroenterology and Hepatology, Changi General Hospital, Singapore

Perforation during esophageal endoscopic submucosal dissection (ESD) can lead to severe complications; localized muscle defects can result in large perforations during ESD that require surgery 1 . While the effectiveness of endoclips or over-the-scope clip systems (OTSC; Ovesco Endoscopy AG, Tübingen, Germany) for the closure of small perforations is well recognized, there is a limit to the size of defect they can close 2 . Moreover, OTS clips may lead to stenosis 3 . Nomura et al. 4 developed a reopenable-clip over-the-line method (ROLM) for complete closure of mucosal defects after ESD. ROLM requires reopenable endoclips with an opening in the jaw (SureClip, 16 mm; ROCC-F-26–195-C; Microtech, Nanjing, China) and a nylon line (0.22 mm), which is also useful in muscular defect closure 5 . Herein, we report a case in which a large perforation that occurred during esophageal ESD was successfully closed using ROLM.

A 75-year-old man underwent ESD for a 12-mm superficial esophageal tumor in the middle esophagus, diagnosed as T1a (epithelium or lamina propria mucosa) ( Fig. 1 ). A large perforation occurred during the submucosal dissection, suggesting the presence of unexpected muscle defects immediately deep to the lesion ( Fig. 2 , Fig. 3 ). After complete mass resection, the perforation was closed using ROLM ( Fig. 4 , Video 1 ) and the patient was treated conservatively with fasting, antibiotics, and a nasogastric tube, without the need for emergency surgery after the ESD. On postoperative day (POD) 6, repeat endoscopy revealed a healing esophageal ulcer with a deep perforation, which on POD 10 had resolved ( Fig. 5 ). An oral diet was resumed on POD 13 and the patient was discharged on POD 17. Follow-up endoscopy did not reveal stricture formation.

**Fig. 1:**
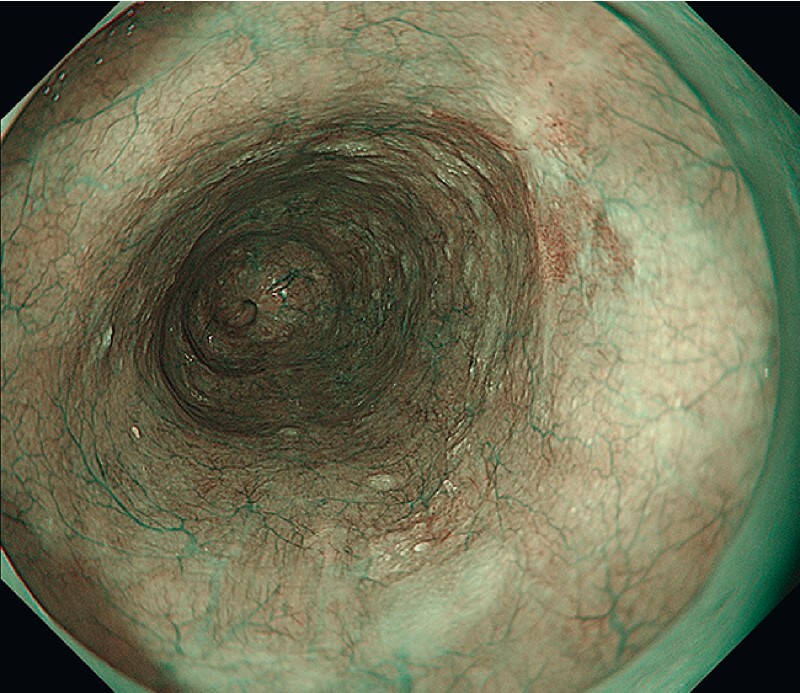
A 12-mm squamous cell carcinoma tumor in the middle esophagus.

**Fig. 2:**
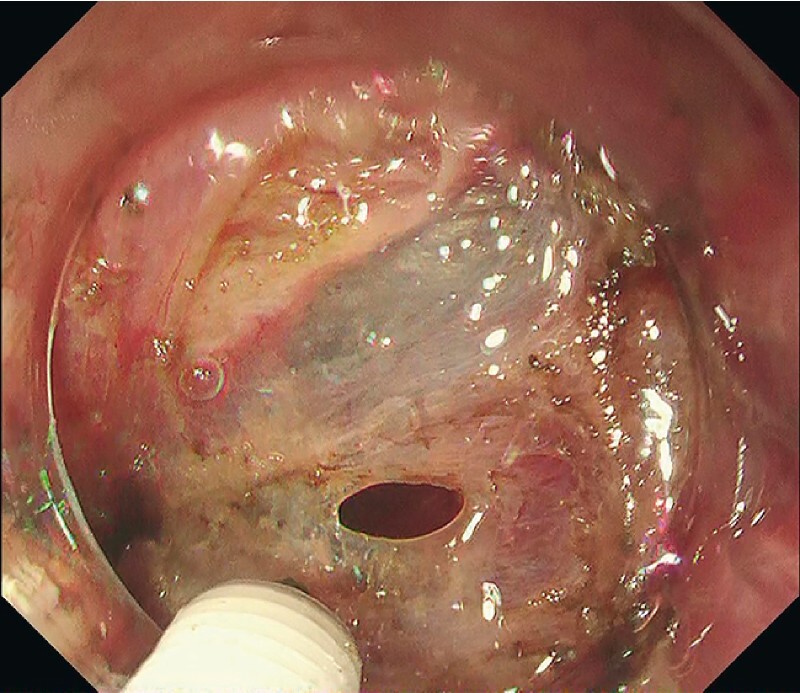
An esophageal muscular layer defect is seen, and a small perforation is observed.

**Fig. 3:**
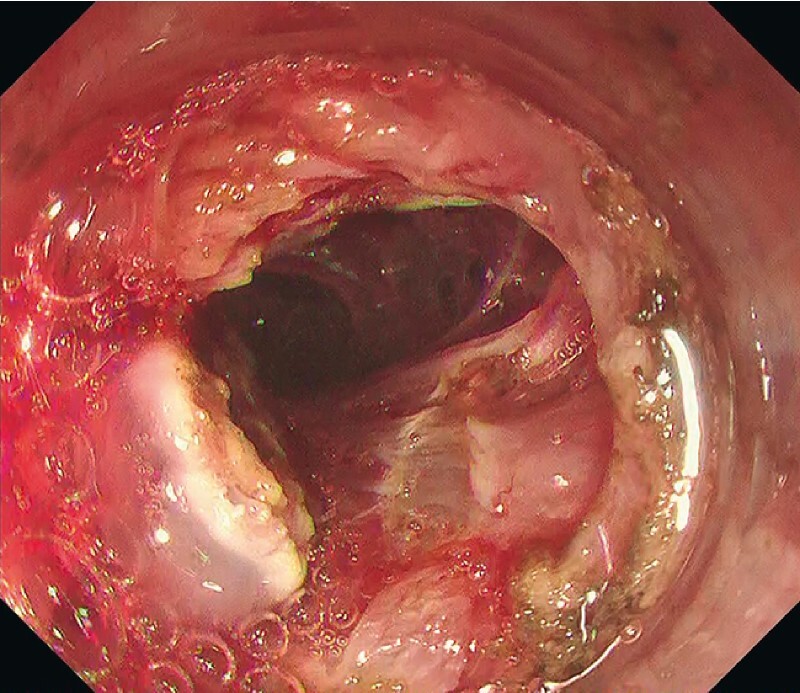
A large perforation occurred during submucosal dissection, suggesting the presence of unexpected muscle defects immediately deep to the lesion.

**Fig. 4:**
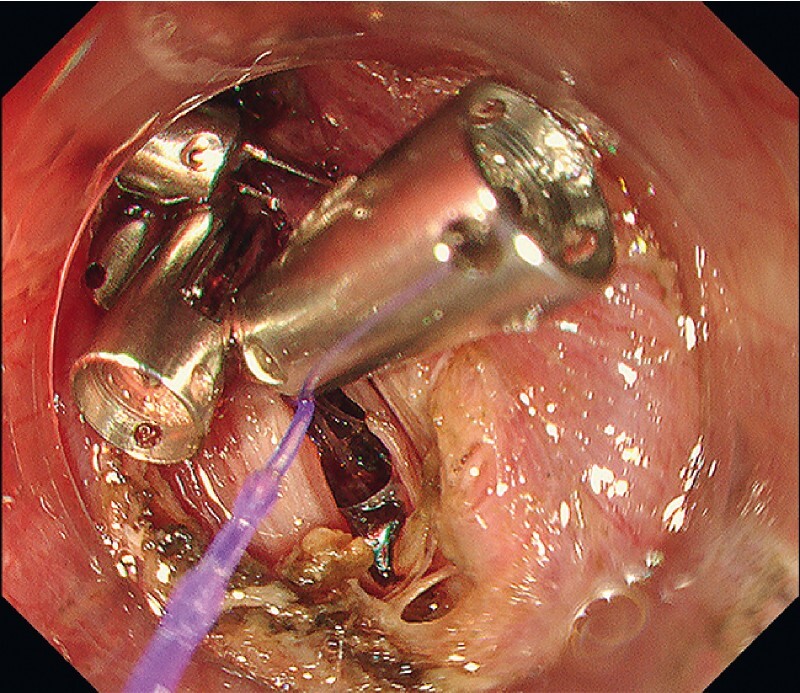
The perforation was closed with repeated use of the reopenable-clip over-the-line method.

**Video 1** Reopenable-clip over-the-line method for closure of large perforation during esophageal endoscopic submucosal dissection.


**Fig. 5:**
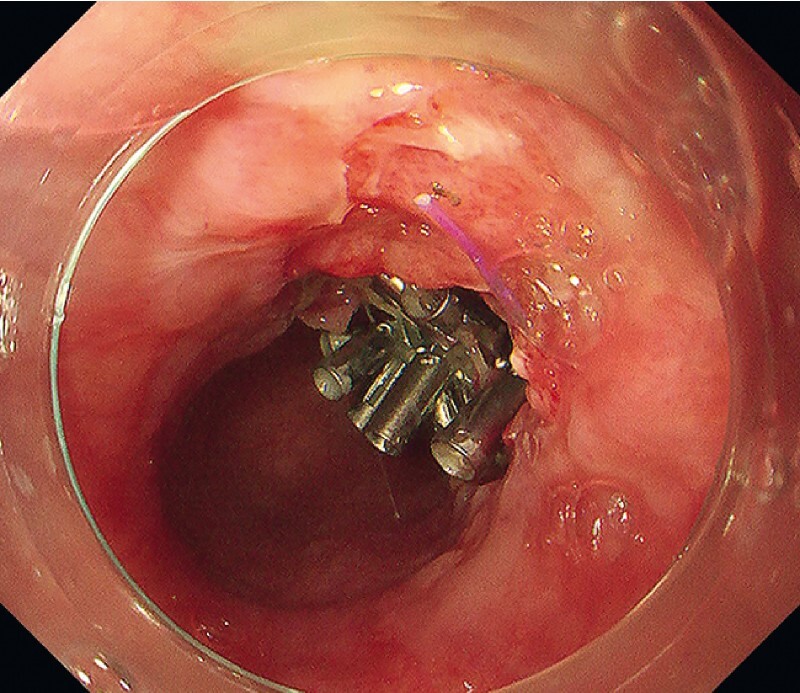
Post–endoscopic submucosal dissection wound at postoperative day 10.

Localized muscle defects may be present in the esophagus and can result in unexpectedly large perforations during ESD. In such cases, ROLM may be a useful endoscopic treatment option to avoid invasive surgery.

Endoscopy_UCTN_Code_TTT_1AO_2AI

## Notes

Endoscopy E-Videos

https://eref.thieme.de/e-videos
*E-Videos* is an open access online section of the journal *Endoscopy* , reporting on interesting cases and new techniques in gastroenterological endoscopy. All papers include a high-quality video and are published with a Creative Commons CC-BY license. Endoscopy E-Videos qualify for HINARI discounts and waivers and eligibility is automatically checked during the submission process. We grant 100% waivers to articles whose corresponding authors are based in Group A countries and 50% waivers to those who are based in Group B countries as classified by Research4Life (see: https://www.research4life.org/access/eligibility/ ). This section has its own submission website at https://mc.manuscriptcentral.com/e-videos
